# A mechanistic investigation exploring the differential transfection efficiencies between the easy-to-transfect SK-BR3 and difficult-to-transfect CT26 cell lines

**DOI:** 10.1186/s12951-017-0271-8

**Published:** 2017-05-02

**Authors:** Elizabeth Figueroa, Pallavi Bugga, Vishwaratn Asthana, Allen L. Chen, J. Stephen Yan, Emily Reiser Evans, Rebekah A. Drezek

**Affiliations:** Rice University, 6100 Main Street, Houston, TX 77005 USA

**Keywords:** Transfection, PEI, AuPAMAM, Autophagy

## Abstract

**Background:**

Gold–polyamidoamine (AuPAMAM) has previously been shown to successfully transfect cells with high efficiency. However, we have observed that certain cell types are more amenable to Au–PAMAM transfection than others. Here we utilized two representative cell lines—a “difficult to transfect” CT26 cell line and an “easy to transfect” SK-BR3 cell line—and attempted to determine the underlying mechanism for differential transfection in both cell types. Using a commonly established poly-cationic polymer similar to PAMAM (polyethyleneimine, or PEI), we additionally sought to quantify the relative transfection efficiencies of each vector in CT26 and SK-BR3 cells, in the hopes of elucidating any mechanistic differences that may exist between the two transfection vectors.

**Results:**

A comparative time course analysis of green fluorescent protein reporter-gene expression and DNA uptake was conducted to quantitatively compare PEI- and AuPAMAM-mediated transfection in CT26 and SK-BR3, while flow cytometry and confocal microscopy were used to determine the contribution of cellular uptake, endosomal escape, and cytoplasmic transport to the overall gene delivery process. Results from the time course analysis and flow cytometry studies revealed that initial complex uptake and cytoplasmic trafficking to the nucleus are likely the two main factors limiting CT26 transfectability.

**Conclusions:**

The cell type-dependent uptake and intracellular transport mechanisms impacting gene therapy remain largely unexplored and present a major hurdle in the application-specific design and efficiency of gene delivery vectors. This systematic investigation offers insights into the intracellular mechanistic processes that may account for cell-to-cell differences, as well as vector-to-vector differences, in gene transfectability.

**Electronic supplementary material:**

The online version of this article (doi:10.1186/s12951-017-0271-8) contains supplementary material, which is available to authorized users.

## Background

Gene transfection is a widely used technique in fundamental and translational biomedical sciences that involves the introduction of foreign genetic material into cells for the purposes of gene modification or therapy. Transfection methods can traditionally be classified into two categories: viral and non-viral. In the former case, genetically modified viral vectors—such as retroviruses or adenoviruses—are used to protect and deliver DNA into cells. While these types of vectors are able to achieve high transfection yields, they are often limited in the size of the genetic payload that can be delivered, their cost of production, and their inherent immunogenicity and oncogenicity [1, 2]. To address these limitations, synthetic non-viral vectors are generally used as an alternative transfection method, as they are less expensive to produce, more scalable, and less immunogenic than their viral counterparts [1, 3, 4]. Given these advantages, non-viral vectors have recently gained increasing prevalence in a number of in vitro and in vivo gene delivery applications [5–8]. In this study, we focus solely on the mechanisms of non-viral vectors, specifically gold–polyamidoamine (AuPAMAM) and polyethyleneimine (PEI).

Most non-viral vectors self-assemble with DNA through electrostatic interactions, forming vector/DNA complexes. These complexes are generally shuttled into cells by receptor-mediated or adsorptive endocytosis, after which they are trafficked through the endosomes then transported from the cytoplasm to the nucleus following endosomal escape. Once in the nucleus, the vector/DNA complexes dissociate, and the DNA gets transcribed [3].

Transfection efficiency is influenced by many factors, including the chemical properties of the vector, the mechanism of uptake, and intracellular delivery routes. Different classes of vectors suffer at different phases of the gene delivery process. For example, non-viral vectors are frequently limited in their ability to navigate the dense cytoplasm and reach the nucleus [9]. Further, independent of the vector chosen, cell types also vary widely in their ability to be transfected [10–13]. The cell type-dependent uptake and intracellular transport mechanisms impacting gene therapy remain largely unexplored and present a major hurdle in the application-specific design and efficiency of gene delivery vectors. In order to investigate the underlying mechanisms responsible for producing cell-to-cell and vector-to-vector differences in gene transfectability, we applied gold-polyamidoamine (AuPAMAM) nanoparticle vectors and polyethylenimine (PEI) vectors for the delivery of DNA in a so-called “easy to transfect” (SK-BR3 human breast adenocarcinoma) and “difficult to transfect’ (CT26 murine colon carcinoma) cell line [14, 15]. The transfection efficiency of both cell lines was quantified, and vector/DNA complex uptake, endosomal escape, and intracellular trafficking investigated using flow cytometry, fluorescence confocal microscopy, and cellular transmission electron microscopy (TEM).

## Results

### Differential transfection in SK-BR3 and CT26

**Fig. 1:**
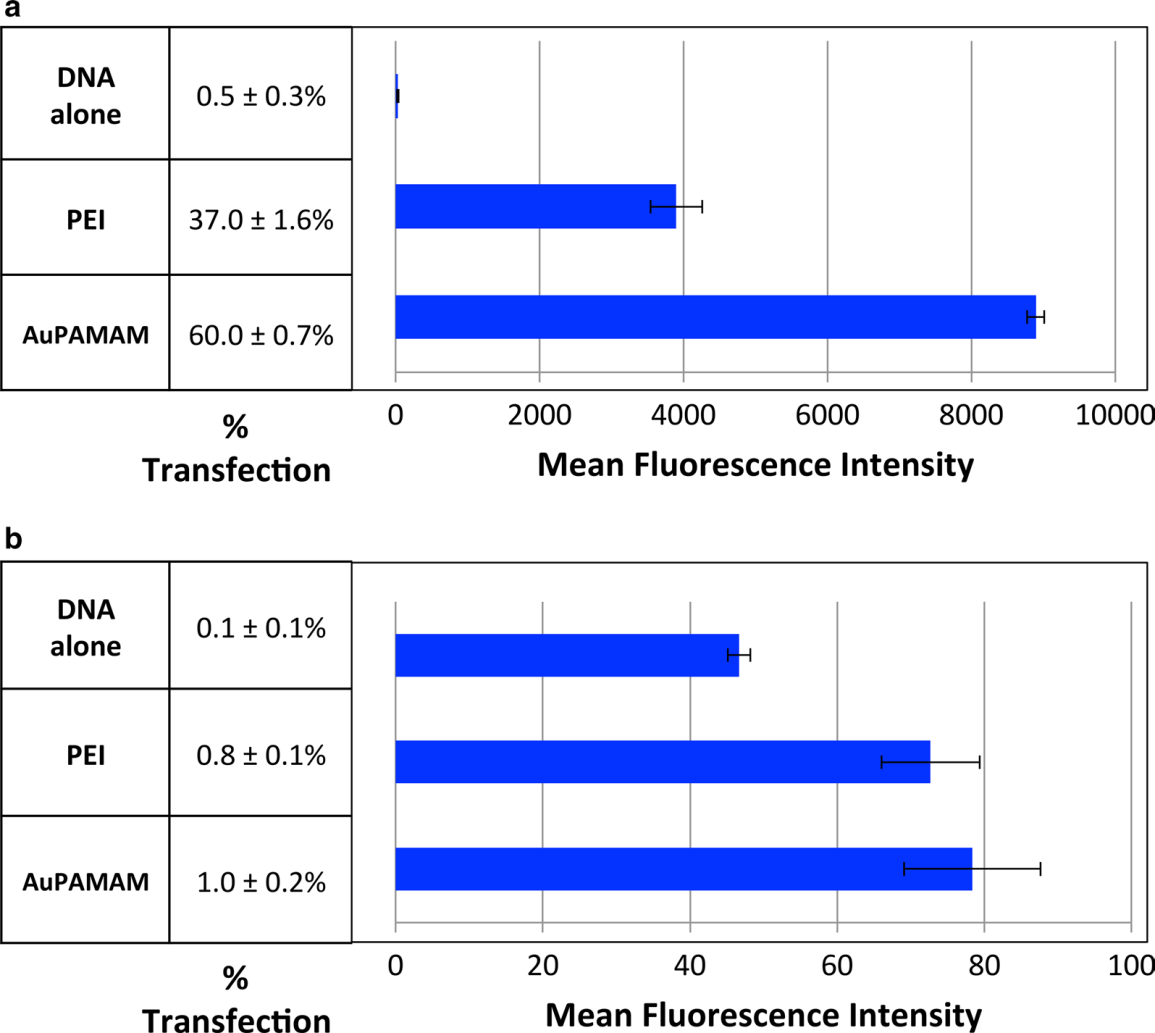
Differential transfection efficiencies of SK-BR3 and CT26 cells. GFP reporter gene plasmids were delivered into both **a** SK-BR3 and **b** CT26 cells either uncomplexed (DNA alone), complexed to PEI, or complexed to AuPAMAM. SK-BR3 cells were given 48 h for incubation, while CT26 cells received 72 h. Transfection efficiency was measured via quantification of percent transfection and mean fluorescent intensity (MFI)

To establish the differential transfection efficiency of SK-BR3 and CT26 cells, a green fluorescent protein (GFP) reporter gene was delivered into both cell lines either alone (no vector), as PEI/DNA, or as AUPAMAM/DNA. The differential transfection efficiency was then quantified using flow cytometry on the basis of two metrics: percent transfection, and mean fluorescence intensity (MFI). Percent transfection corresponds to the percentage of cells that have been successfully transfected, and thus fluoresce green. Mean fluorescence intensity, on the other hand, is a measure of the intensity of GFP expression per cell, and corresponds to the extent by which each cell has been transfected. In Fig. 1, the results of transfection with DNA alone (no vector), PEI/DNA, and AuPAMAM/DNA are shown for both SK-BR3 cells and CT26 cells following a 48 and 72 h transfection period respectively. Note that CT26 cells were given an additional 24 h of transfection as no fluorescence was observed at the 48-h time point. In the “easy to transfect” SK-BR3 cell line, PEI and AuPAMAM both performed significantly better than DNA alone in terms of percent transfection (37 and 60% versus 0.5%, respectively) and MFI (3902 and 8896 versus 32 fluorescence intensity units, respectively). In the “difficult to transfect” CT26 cell line, PEI and AuPAMAM performed only marginally better than DNA alone in terms of percent transfection (0.8 and 1.0% versus 0.1%, respectively) and MFI (73 and 78 versus 47 fluorescence intensity units, respectively).

### Intracellular DNA uptake

**Fig. 2:**
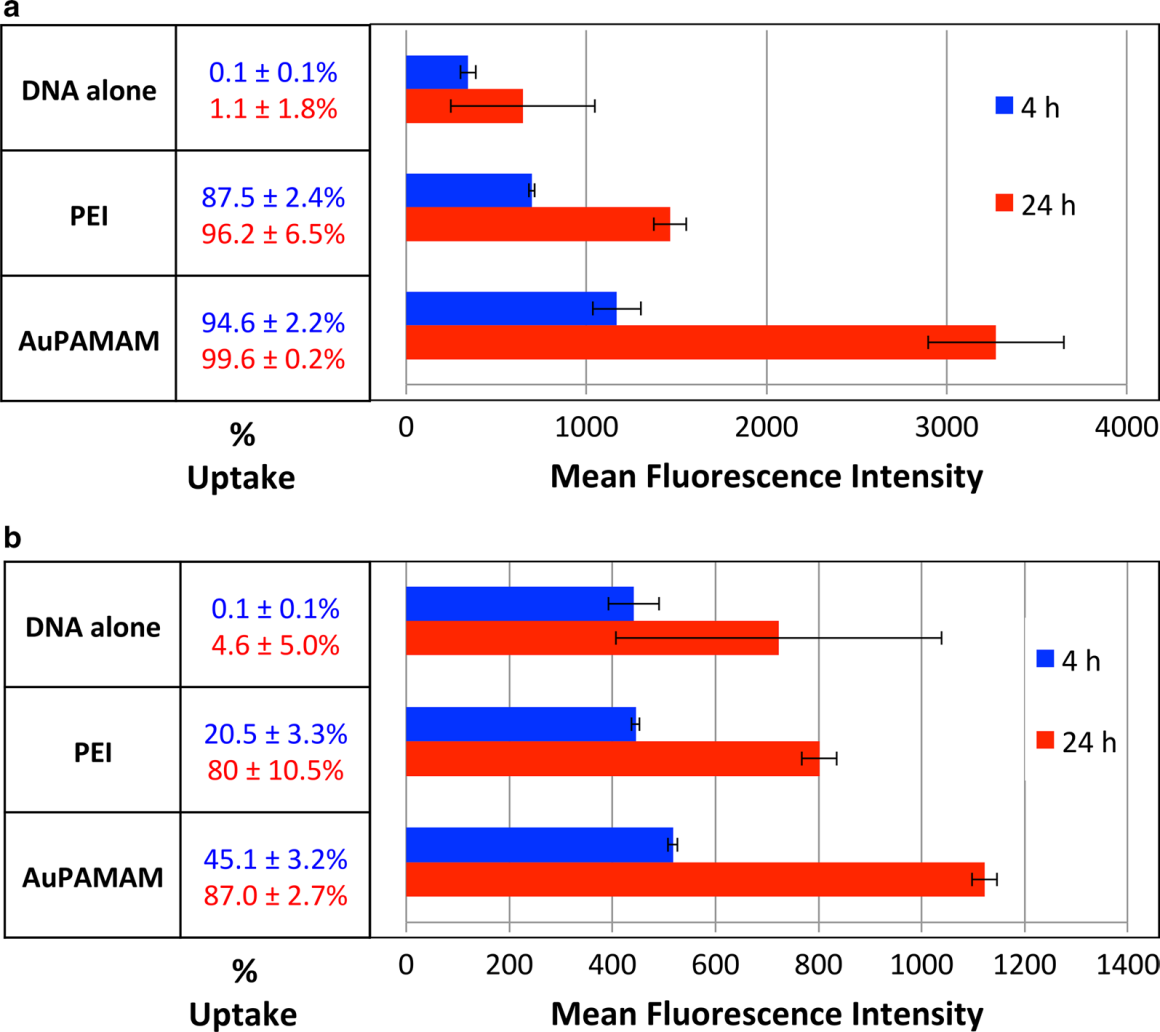
Percent uptake of Cy5-labeled DNA in SK-BR3 and CT26 cells. Cy5-labeled DNA was delivered into both **a** SK-BR3 and **b** CT26 cells either uncomplexed (DNA alone), complexed to PEI, or complexed to AuPAMAM, and allowed to incubate for 4 and 24 h before being analyzed via flow cytometry. Uptake efficiency was measured via quantification of percent uptake and mean fluorescent intensity (MFI)

To determine whether DNA uptake produces the differential transfection efficiencies observed in SK-BR3 and CT26, Cy5-labeled DNA was delivered into both cell lines; following analysis of both cell lines using flow cytometry, percent uptake and MFI were quantified. Figure 2 shows the percent uptake (percentage of cells that contain Cy5-labeled DNA) and the MFI of Cy5 fluorescence per cell. Four-hour and 24-h time points were chosen as they represent early and late stages of transfection. At 4 h post transfection, the percent uptake with PEI is significantly greater in SK-BR3 than in CT26 (87.5 versus 20.5%). A similar trend is seen with the percent uptake of SK-BR3 and CT26 cells using AuPAMAM (94.6 versus 45.1%, respectively). At 24 h however, the percentage of Cy5 uptake in CT26 cells increases to nearly match that of SK-BR3 cells for both PEI (80.0 versus 96.2%) and AuPAMAM (87 versus 99.6%). Despite this increase, the MFI at 24 h is still markedly greater in SK-BR3 cells compared to CT26 cells for both PEI (1464 versus 802 fluorescence intensity units) and AuPAMAM (3274 versus 1122 fluorescence intensity units). This indicates that the SK-BR3 cells are taking up larger quantities of Cy5-labeled DNA per cell than CT26 cells.

### Endosomal escape

**Fig. 3:**
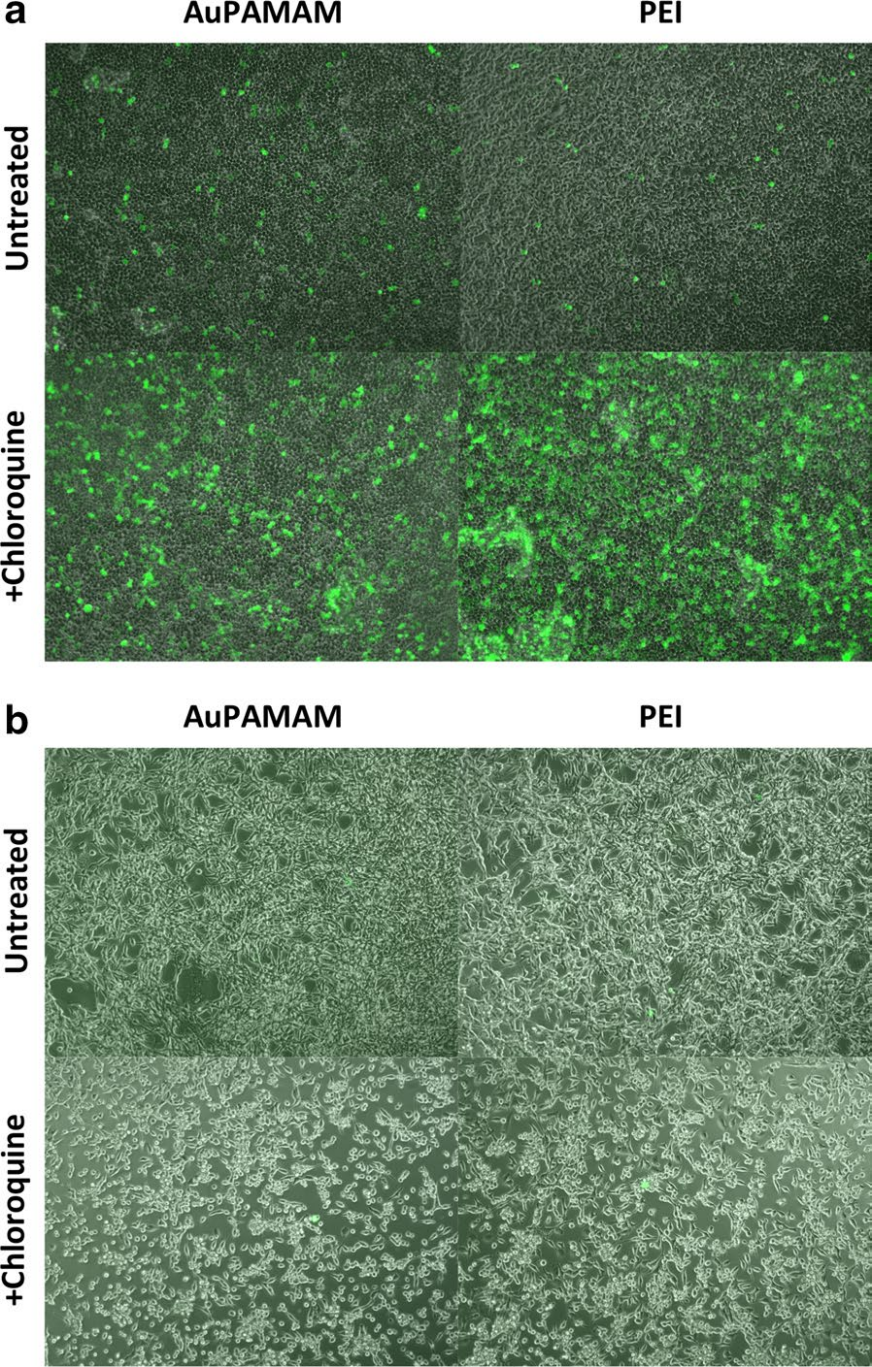
Effects of chloroquine on endosomal escape in SK-BR3 and CT26 cells. GFP reporter gene plasmids were delivered into **a** SK-BR3 and **b** CT26 cells either complexed to AuPAMAM or PEI. Cells were treated with 1000 μM chloroquine, and allowed to incubate for 3 h. Enhancement of endosomal escape and overall transfection efficiency by chloroquine was then visualized by fluorescence microscopy. Images shown are fluorescent images overlaid on transmission images

In order to investigate whether endosomal escape plays an important role in producing the differential transfection efficiencies observed in CT26 and SK-BR3, both cell lines were treated with chloroquine, a common lysosomotropic agent that accumulates preferentially in acidic organelles (endosomes and lysosomes) and causes membrane rupture via inhibition of acidification and subsequent osmotic swelling. Enhancement of transfection efficiency by chloroquine was quantified using a GFP reporter gene. As shown in Fig. 3, when treated with chloroquine, SK-BR3 cells exhibited enhanced transfection for both the PEI and AuPAMAM vectors, as made evident by the increased fluorescent signal produced by these cell populations. Though the AuPAMAM condition displayed greater GFP fluorescence than PEI in the absence of chloroquine, PEI produced greater GFP fluorescence than AuPAMAM in the presence of chloroquine. In contrast, CT26 cells displayed no significant improvement in transfection efficiency when treated with chloroquine for either PEI or AuPAMAM. This is shown clearly in Fig. 3, as there is no discernable increase in GFP fluorescence between the untreated and treated conditions.

### Subcellular trafficking of DNA with confocal imaging

**Fig. 4:**
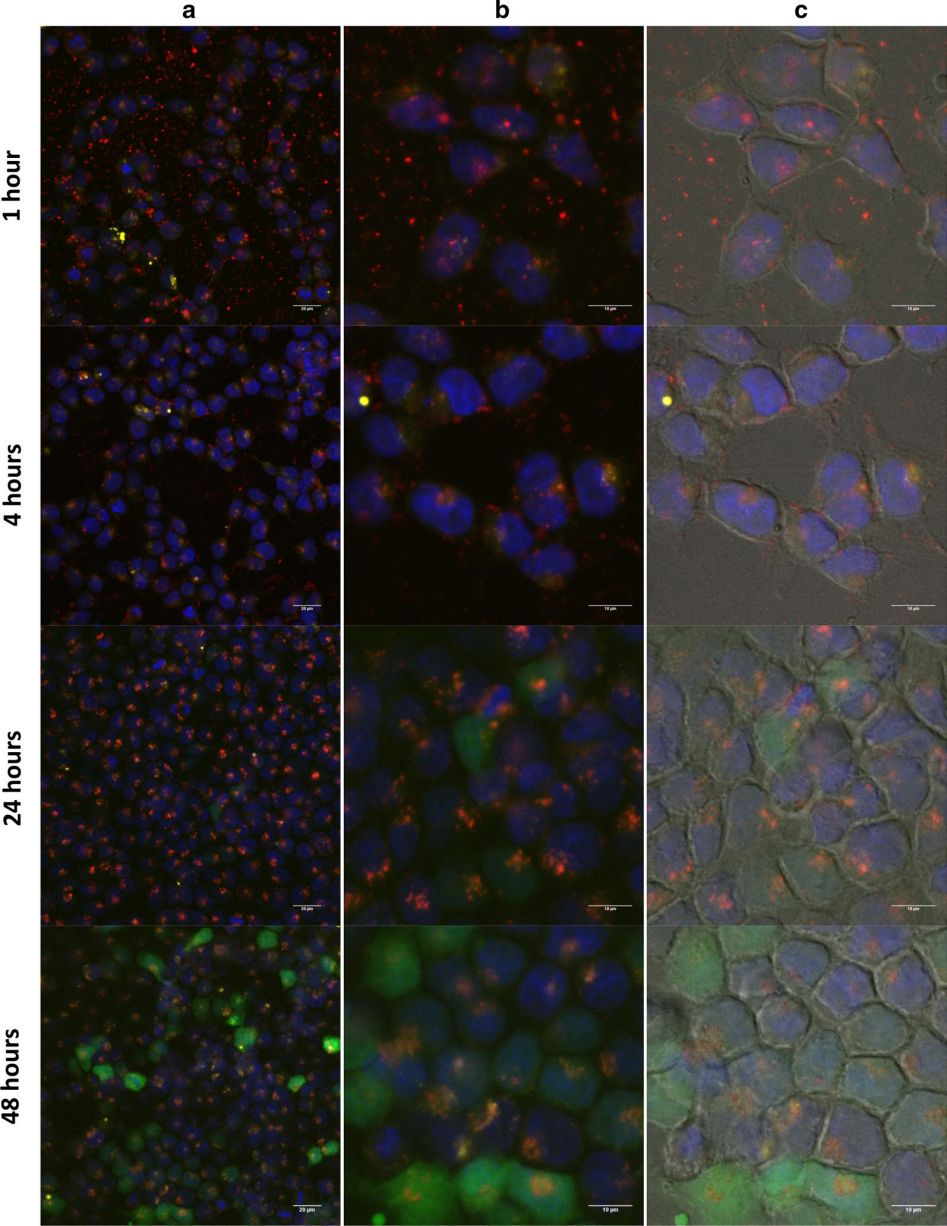
Subcellular trafficking of Cy5-labeled AuPAMAM/DNA complexes in SK-BR3 cells. **a** The intracellular trafficking of Cy5-labeled GFP reporter gene plasmid DNA (shown in *red*) was observed in SK-BR3 cells 1-, 4-, 24-, and 48-h post-transfection via confocal microscopy. Column (**a**) depicts 60× magnification and column (**b**) depicts Nyquist zoom of the corresponding images in column (**a**). Column (**c**) exhibits overlays of the fluorescent channels with the transmission channel for the corresponding images in column (**b**). *Scale bar* is (**a**) 20 μm and (**b**, **c**) 10 μm

**Fig. 5:**
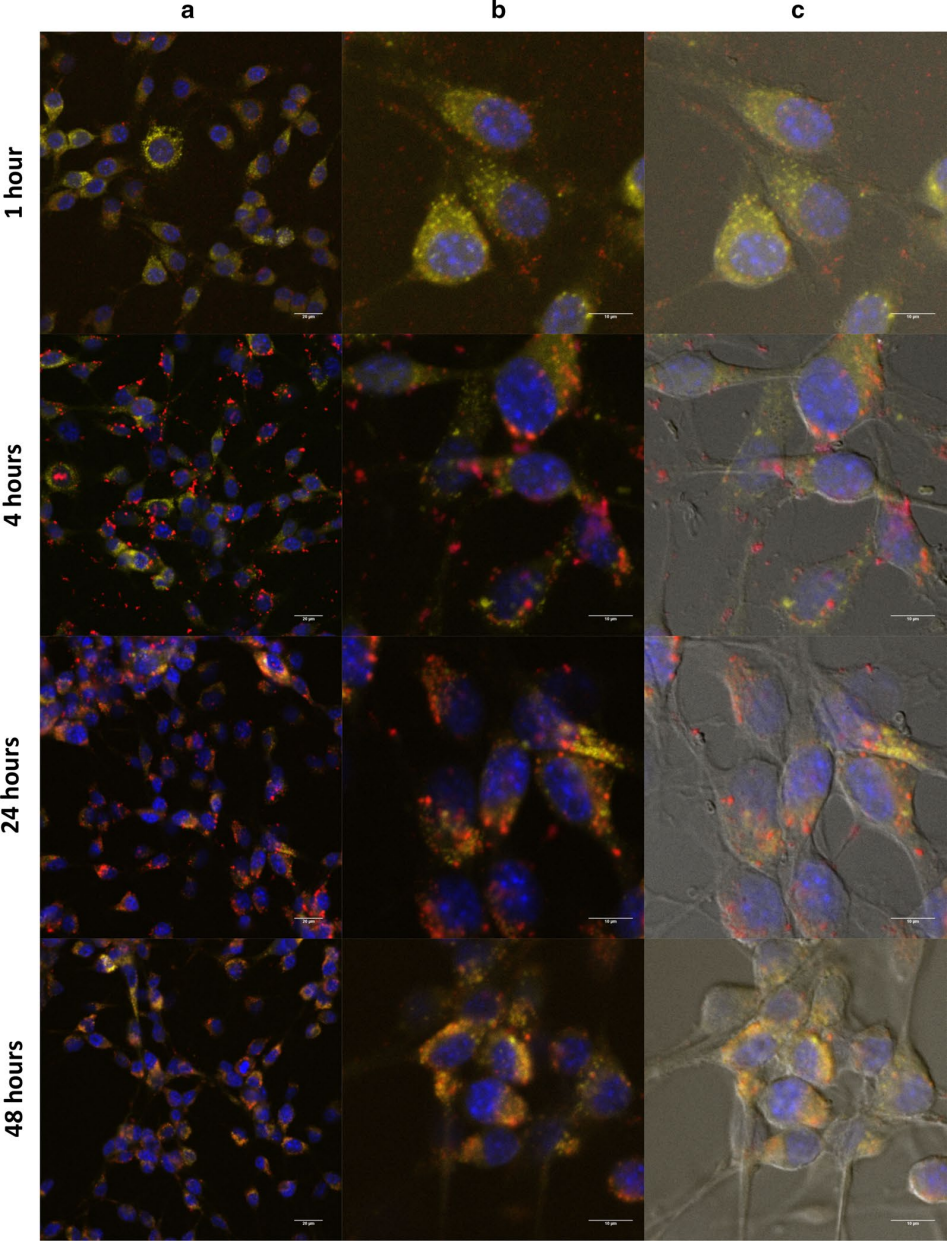
Subcellular trafficking of Cy5-labeled AuPAMAM/DNA complexes in CT26 cells. **a** The intracellular trafficking of Cy5-labeled GFP reporter gene plasmid DNA (shown in *red*) was observed in CT26 cells 1-, 4-, 24-, and 48-h post-transfection via confocal microscopy. Column (**a**) depicts 60× magnification and column (**b**) depicts Nyquist zoom of the corresponding images in column (**a**). Column (**c**) exhibits overlays of all fluorescent channels with the transmission channel for the corresponding images in column (**b**). *Scale bar* is (**a**) 20 μm and (**b**, **c**) 10 μm

Having studied the role of endosomal escape in producing the differential transfection efficiencies observed in SK-BR3 and CT26, we attempted to follow the trafficking of DNA intracellularly. To monitor the intracellular trafficking of plasmid DNA during transfection, subcellular localization of Cy5-labeled DNA was observed via confocal and bright field microscopy (Figs. 4, 5). Cells were imaged at 1, 4, 24, and 48 h post-transfection. Nuclei were stained by DAPI (shown in blue) while lysosomes and other acidic organelles were stained with Lysotracker Yellow (shown in yellow). Though experiments were conducted for both AuPAMAM and PEI, here, we shall focus solely on the results of AuPAMAM transfection; the results of PEI transfection can be found in Additional file 1: Figure S1 and Additional file 2: Figure S2.

Subcellular trafficking of AuPAMAM/DNA complexes was first analyzed in SK-BR3 cells (Fig. 4). In the 1-h condition, numerous red fluorescent spots (representing Cy5-labeled DNA) are seen within the cells, suggesting that many AuPAMAM/DNA complexes have already been internalized. Slight co-localization of the Cy5-labeled DNA with Lysotracker Yellow is observed in a few of the cells, as is evident by the overlapping red and yellow fluorescent signals. A number of red fluorescent spots are also visible outside of the cell border, as is shown in the bright field images taken 1-h post transfection (Fig. 4c). However, given that the cells were washed following the 1-h incubation, and that no other time points exhibit such extracellular fluorescent signals, we can likely conclude that such extracellular fluorescence is due to incomplete washing of these cells. At 4 h post-transfection, co-localization of DNA with the Lysotracker becomes increasingly apparent, indicating that more particles have localized within acidic organelles (endosomes or lysosomes). Red fluorescent spots are also visible in almost every cell, suggesting that the percent uptake of the AuPAMAM/DNA complexes is increasing.

At the 24-h time point, there is a marked increase in the number of red fluorescent spots seen within each cell, signifying that more AuPAMAM/DNA complexes have been internalized per cell. These results more or less corroborate those observed in Fig. 2, as the percent transfection remains approximately the same between the 4- and 24-h conditions, while the MFI increases in the latter. At 24-h post transfection, there also appears to be co-localization of the DNA with DAPI, suggesting that some of the DNA has already traversed to and localized within the nucleus. Transcription and translation of the DNA has also clearly begun by this point, given that green fluorescence is detectable in a few of the cells. By 48 h, more cells appear to be expressing the GFP reporter gene, as green fluorescence is largely apparent in at least half of the cells. This result is in agreement with the percent transfection observed in Fig. 1 (60%). Punctate clusters of DNA, appearing as small rings of red fluorescent spots, are also visible in or adjacent to the nuclei of almost every cell at this time point.

Subcellular trafficking was next analyzed in CT26 cells (Fig. 5). At 1 h post-transfection, only a small percent of cells appear to have internalized the DNA complexes, as evident by the lack of a distinct red fluorescent signal in a number of the cells. Yet, co-localization of DNA with the Lysotracker is detectable in those cells that have taken up the AuPAMAM/DNA complexes. At 4 h post-transfection, more cells have begun to take up the AuPAMAM complexes; however, the presence of numerous red fluorescent spots near the peripheries of many of the cell membranes indicates that the process of uptake is likely still ongoing at this time point. As was observed at 1 h post transfection, co-localization of DNA with Lysotracker Yellow is again visible in the 4-h condition, albeit much more apparent. This implies that the AuPAMAM/DNA complexes have already localized, or begun to localize, within acidic organelles. By 24 h, almost all cells appear to have internalized the AuPAMAM/DNA complexes, thus qualitatively confirming the Cy5-labeled DNA uptake observed in Fig. 2 (87%) for AuPAMAM in CT26. Unlike SK-BR3 however, the CT26 cells do not show any localization of DNA within the nuclei at this time point. Rather, most of the DNA appears to remain confined in acidic organelles. Finally, at 48 h post-transfection, DNA continues to co-localize with Lysotracker, and co-localization of DNA with DAPI is not observed. Most of the complexes appear to have migrated internally away from the cell membranes. Additionally, no GFP fluorescence is detected in any of the cells, therefore confirming the almost negligible percent transfection observed in Fig. 1 (1.0% for AuPAMAM in CT26). For both the 24- and 48-h conditions, blebbing and irregular cell morphologies are also observed.

To determine whether AuPAMAM remains complexed to the DNA over the whole duration of transfection, AuPAMAM was first imaged in transmission mode (to visualize the gold nanoparticles that form the core of AuPAMAM vectors), then subsequently merged with the fluorescent channel displaying Cy5 signal from Cy5-labeled DNA (Additional file 3: Figure S3). Results from this experiment revealed significant co-localization of AuPAMAM with DNA 24 h post-transfection in both SK-BR3 and CT26, as demonstrated by the overlap observed between the fluorescent regions (in the red channel) and dense black regions (in transmission mode).

### Intracellular tracking of AuPAMAM with cellular TEM

To further study the intracellular trafficking pathway of AuPAMAM/DNA complexes and more clearly elucidate the subcellular localization of AuPAMAM particles in various organelles, CT26 cells and SK-BR3 cells were imaged using transmission electron microscopy (TEM) at 1, 4, and 24 h post transfection. Gold nanoparticles (which form the core of AuPAMAM) are electron dense, and thus easy to visualize via TEM. PEI, on the other hand, is far less electron dense, and thus far more difficult to visualize using TEM. For this reason, only AuPAMAM was investigated using this approach.

**Fig. 6:**
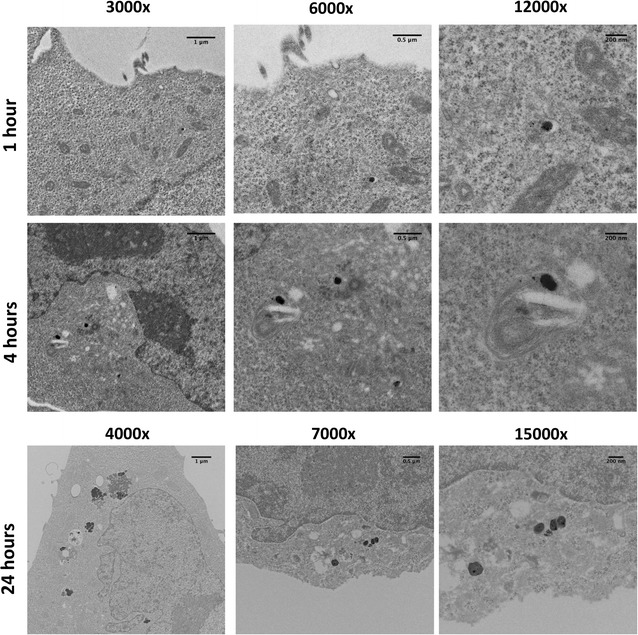
Intracellular trafficking of AuPAMAM/DNA complexes in SK-BR3 cells. The intracellular trafficking of AuPAMAM/DNA complexes was observed in SK-BR3 cells 1-, 4-, and 24-h post-transfection via cellular TEM imaging. *Scale bars* are 1 μm (3000× and 4000×), 0.5 μm (6000× and 7000×), and 200 nm (12,000× and 15,000×)

The results of TEM imaging in SK-BR3 cells is presented first. At 1 h post-exposure, AuPAMAM/DNA complexes have already begun internalizing, as demonstrated by the presence of sub-100 nm complexes within various organelles within the cell (Fig. 6). As seen in the confocal imaging experiment (Fig. 4), there is scarce co-localization of AuPAMAM/DNA complexes within lysosomes at this time-point. At 4 h, the density of AuPAMAM/DNA complexes in intracellular organelles appears to be greater than in the 1-h time-point. Particle internalization was apparent at this time-point as well. At 24-h post transfection, AuPAMAM/DNA complexes are seen clustered peri-nuclearly. Vesicles containing particle complexes also appear to be localized near the periphery of the cell, with cellular morphologies perhaps indicating particle exocytosis.

**Fig. 7:**
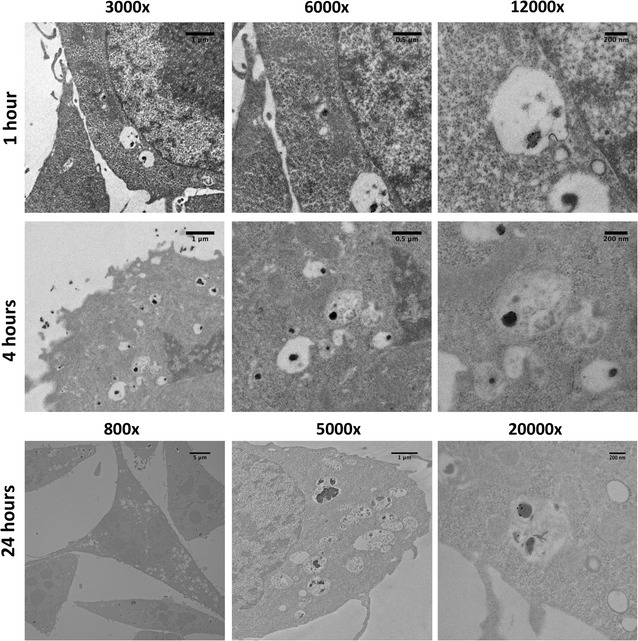
Intracellular trafficking of AuPAMAM/DNA complexes in CT26 cells. The intracellular trafficking of AuPAMAM/DNA complexes was observed in CT26 cells 1-, 4-, and 24-h post-transfection via cellular TEM imaging. *Scale bars* are 5 μm (800×), 1 μm (3000× and 5000×), 0.5 μm (6000×), and 200 nm (12,000× and 20,000×)

The results of the TEM imaging in CT26 cells exhibit a marked departure from those in SK-BR3 (Fig. 7). In the 1-h condition, AuPAMAM/DNA complex internalization is visible, based on the presence of particles within the cell. Analysis via ImageJ also reveals larger complex sizes in CT26 cells versus SK-BR3 at this time-point. At 4 h post-transfection, prevalent membrane ruffling and cytoplasmic extensions are observed. Particle internalization still appears to be continuing on into this time-point as well. Interestingly, as opposed to the complexes observed in SK-BR3 cells, the AuPAMAM/DNA complexes visualized in CT26 cells appear to be predominantly encapsulated in endosome-like cytoplasmic vesicles, with a high density of such structures present within the cell. At the 24-h time-point, the number of these particle-containing cytoplasmic structures increases, and scarce peri-nuclear localization of complexes is observed.

## Discussion

### Differential transfection in SK-BR3 and CT26 cell lines

To establish the respective transfection efficiencies of SK-BR3 and CT26 cells, a GFP reporter gene was delivered either alone (no vector), complexed to PEI, or complexed to AuPAMAM. A comparison of both the percent transfection and MFI of DNA only, PEI/DNA complexes, and AuPAMAM/DNA complexes in SK-BR3 and CT26 indicate that SK-BR3 cells are considerably easier to transfect than CT26, even when CT26 cells are allowed to undergo a 72-h transfection period (as opposed to the 48-h transfection period observed for SK-BR3 cells). As stated previously, percent transfection refers to the number of cells that have been successfully transfected and transcribed, and thus fluoresce green. The fact that SK-BR3 cells have a greater percent transfection than CT26 cells therefore suggests that the SK-BR3 cells may be uptaking complexes, trafficking those complexes, and/or transcribing the DNA more efficiently than CT26 cells. This conclusion is reaffirmed when considering the greater MFI of SK-BR3 cells as compared to CT26. Interestingly, the complexation of DNA with either AuPAMAM or PEI did little to enhance the efficiency of transfection/transcription in CT26 cells, despite the fact that DNA delivered without a vector is generally rapidly degraded by nucleases in the cytoplasm [16]. This observation may further suggest that CT26 transfection is limited by a lack of complex uptake or cytoplasm to nuclear trafficking. To, test the former hypothesis, we first investigated the efficiency of complex uptake in both cell lines.

### Intracellular DNA uptake

Percent transfection and MFI provide metrics for quantifying the efficiency of both transfection and transcription, as they measure the intensity of the fluorescent signal produced from the transcription of GFP within the cell. One issue with using these metrics in this way, however, is that it is difficult to determine whether the discrepancies observed in Fig. 1 between SK-BR3 and CT26 are arising due to differences in transfection efficiency, or differences in transcription. In order to subvert this potential ambiguity, intracellular DNA uptake was investigated by delivering Cy5-labeled DNA, complexed to either PEI or AuPAMAM, into CT26 and SK-BR3. By delivering DNA that is fluorescently labeled, any differences that arise in the overall DNA uptake between the two cell lines can be attributed purely to the processes involved in transfection—such as cellular uptake, endosomal escape, and cytoplasm to nuclear trafficking—rather than those related to transcription.

Quantification of percent uptake and MFI at both 4- and 24-h indicate that complex internalization is slower in CT26 cells compared to SK-BR3, for both PEI and AuPAMAM. Though the percent uptake of both vectors remains low in CT26 (compared to SK-BR3) at the 4-h time point, the percent uptake in CT26 nearly reaches that of SK-BR3 at the 24-h time point. Given that percent uptake corresponds to the percentage of cells that have internalized at least a minimum number of vector/DNA complexes, it can be concluded here that a comparable percentage of CT26 cells and SK-BR3 cells are able to uptake complexes and internalize the Cy5-labeled DNA, albeit at very different rates. A comparison of MFI in both cell types also supports this hypothesis. Though the percent uptake of PEI/DNA and AuPAMAM/DNA complexes is approximately equal in both cell types at the 24-h time point, the MFI of both vectors in SK-BR3 remain significantly greater than those in CT26. This indicates that the rate of internalization in both cell types is varied, as the amount of DNA—or vector/DNA complexes—being taken up by CT26 and SK-BR3 is different. Ultimately, based on the above findings, it can be concluded that CT26 cells are more limited in their ability to uptake both vector/DNA complexes than SK-BR3 cells.

One possible reason for the slower internalization observed in CT26 cells may be due to their overproduction of mucins [17]. Previous transfection studies have shown that mucins may act as a physical barrier to gene delivery and particle uptake due to their propensity to bind to polycationic polyplexes in the local medium and induce aggregate formation [18]. It should be mentioned here that cellular autofluorescence does not appear to be a significant source of variation (in MFI) between the two cell types, given that the DNA only conditions in SK-BR3 and CT26 are approximately same at the 4-h time point (343 versus 441) and 24-h time point (647 versus 722). It is also interesting to note that the DNA alone condition in CT26 is approximately the same as the PEI and AuPAMAM conditions in terms of MFI, but not percent uptake. This result suggests that the presence of a vector increases the probability of DNA to be internalized by a given cell, but does not necessarily enhance the amount of DNA that the given cell will uptake.

When corroborating the results of Figs. 1 and  2, it becomes clear that additional barriers exist beyond complex uptake that are hindering the efficiency of transfection in CT26 cells. In Fig. 2, by the 24-h time point, ~80–90% of CT26 cells have taken up Cy5-labeled DNA for both the PEI and AuPAMAM conditions. However, the percent transfections of PEI and AuPAMAM in Fig. 1 are far below the percent uptakes observed for both vectors in CT26 (~80 versus 1%) despite a 72-h transfection period. Thus, aside from being limited by DNA uptake at early time points, CT26 transfection may also be hindered in the intracellular transport of vector/DNA complexes, especially based on the fact that at later time points, DNA uptake was compensated for, but transfection efficiency still remained low. A comparison of the MFIs of SK-BR3 and CT26 cells in Figs. 1 and  2 further enforces this hypothesis: in Fig. 1, the MFI difference between SK-BR3 and CT26 is 100-fold, whereas in Fig. 2, it is only threefold (even despite the fact that in Fig. 1, CT26 was allowed to incubate for 24 h more than SK-BR3). If uptake were in fact the only barrier affecting the transfection efficiency of CT26 cells, than we would expect the MFI difference in Fig. 1 to be much smaller—roughly around the threefold difference seen with Fig. 2. Taken altogether, transfection efficiency in CT26 may be lowered due to the complexes having (1) difficulty escaping the endo-lysosomal pathway, (2) difficulty trafficking the cytoplasm, or (3) difficulty entering the nucleus.

### Endosomal escape

After successful internalization into cells, vector/DNA complexes must make their way through the endocytic pathway before escaping into the cytoplasm, where they get trafficked to the nucleus. Certain classes of polyplexes, however, can remain stuck in the endocytic pathway, ultimately inhibiting their ability to successfully deliver their genetic payload to the nucleus. To determine whether endosomal escape plays a role in producing the differential transfection efficiencies observed between SK-BR3 and CT26 cells, chloroquine was added to both cell lines. The results observed from the addition of chloroquine indicate that (1) chloroquine appears to enhance the transfection efficiency of both PEI and AuPAMAM in SK-BR3 cells, and (2) PEI may be more limited by endosomal escape in SK-BR3 than AuPAMAM, as it exhibits a greater increase in fluorescent signal following treatment with chloroquine. Taken together, these results suggest that endosomal escape may be an important factor that can be addressed, especially when using a PEI vector, for increased transfection in the SK-BR3 cell line.

Unlike SK-BR3, CT26 cells displayed no significant improvement in transfection efficiency when treated with chloroquine for either PEI or AuPAMAM vectors. One possible explanation for this observation may be due to the redundant pathways exhibited by chloroquine and PEI or AuPAMAM. According to the popular “proton-sponge” hypothesis, certain polycationic vectors, especially those with a high density of amine groups on their surface (such as PEI and AuPAMAM) can act as a buffer in the lysosomes and sequester protons on their amine groups, causing lysosomal alkalinization. In order to combat this increase in lysosomal pH, protons are rapidly pumped into the lysosomes by the vacuolar H+-ATPase. Similar to chloroquine, this ultimately leads to osmotic swelling and lysosomal membrane rupture [19]. Therefore, given the mechanistic redundancies between chloroquine and PEI/AuPAMAM, enhancement of transfection efficiency is not always observed when the two are administered in combination.

The question still remains however as to why SK-BR3 cells experience an enhancement in transfection efficiency in the presence of chloroquine, but CT26 cells do not. One possible explanation for this observation may be due to the exocrine activity of CT26. As stated previously, mucins (which are produced in high concentration by CT26 cells) can often induce increased aggregation of polyplexes by disrupting the electrostatic interactions between the polycationic vector and polyanionic DNA [18]. Previous studies have shown that polyplexes containing higher concentrations of primary amines on their surface can produce a greater degree of alkalinization in the lysosome, generate larger and more permanent ruptures in the lysosomal membrane, and induce increased cellular cytotoxicity [19]. Thus, the interactions of mucins with PEI/DNA and AuPAMAM/DNA in CT26 cells may be causing these complexes to aggregate to a point that is adequate to produce sufficient lysosomal membrane permeabilization for particle release even in the absence of chloroquine. Though this hypothesis requires further testing, the fact that chloroquine failed to improve the transfection efficiency of CT26 cells likely indicates that endosomal escape is not a significant obstacle in the transfection pathway of CT26.

### Subcellular trafficking of vectors with confocal imaging

Having investigated the role of endosomal escape in the transfection pathways of both CT26 and SK-BR3, we next set out to examine the subcellular trafficking of AuPAMAM/DNA and PEI/DNA complexes via imaging of Cy5-labeled DNA using confocal and bright field microscopy. Images were taken at 1, 4, 24 and 48 h post-transfection. Over the 48-h time period, SK-BR3 cells exhibited more rapid complex uptake and cytoplasmic trafficking than CT26, as evidenced by the presence of red fluorescent DNA inside SK-BR3 cells at earlier time points, and the co-localization of DNA with SK-BR3 nuclei at the 24 and 48-h time points. This observation supports our earlier conclusion that CT26 cells may be more limited in vector/DNA complex uptake than SK-BR3 cells.

It is interesting to note that in transfected CT26 cells, almost the entire cytoplasm fluoresces bright yellow when exposed to the Lysotracker dye; this is in direct contrast to SK-BR3, in which only a small portion of the cell’s cytoplasm exhibits a weak yellow fluorescence. These distinct staining patterns appear to indicate a preponderance of acidic organelles within the cytoplasms of transfected CT26 cells relative to SK-BR3 cells. It should also be mentioned that the distribution of DNA also appears to be different in each of the cell types. In SK-BR3 cells, the DNA remains tightly clustered within the nucleus, as made clear by the co-localization of red fluorescent signals with blue DAPI stained nuclei observed in the 24- and 48-h conditions. In CT26 cells however, the DNA appears diffuse within the cytoplasm, with significant co-localization of red fluorescent clusters observed with the Lysotracker; this may implicate cytoplasmic to nuclear trafficking as a potential barrier hindering the transfectability of CT26 cells. The co-localization of Cy5-labeled DNA with Lysotracker observed in CT26 cells likely indicates that the DNA or DNA complexes are being encapsulated by acidic cytoplasmic organelles at some point along their transfection pathway. Quantification (via ImageJ) of the intensity and size of the Cy5 fluorescent clusters observed in both cell lines demonstrate a stronger signal and larger cluster size in CT26 cells as compared to SK-BR3. The increased signal and size of the Cy5 fluorescent spots that are co-localized with the Lysotracker Yellow in CT26 may indicate increased aggregation of the AuPAMAM/DNA complexes, thus potentially validating our previous hypothesis regarding the propensity of mucin to induce such particle interactions.

To determine whether AuPAMAM remains complexed to DNA over the whole course of transfection, transmission images of AuPAMAM were merged with the confocal images of Cy5-labeled DNA. Based on these merged images (shown in Additional file 3: Figure S3), the AuPAMAM/DNA complexes appear to remain intact in both SK-BR3 and CT26, even after 24 and 48-h incubation periods. From the images, it is unclear however whether DNA and AuPAMAM enter the nucleus together, or whether the DNA dissociates from the vector prior to nuclear entry. Further investigation of this topic may provide useful insight into the mechanisms that promote nuclear localization and uptake for polycationic non-viral vectors.

### Intracellular tracking of AuPAMAM with cellular TEM

To further explore the intracellular trafficking of particles in SK-BR3 and CT26, cellular TEM was used to visualize the transfected AuPAMAM/DNA complexes over three time-points. The results of the TEM images for both cell types confirm what was seen previously with the confocal imaging experiments (Figs. 4, 5).

In the 1- and 4-h post-transfection conditions, most of the AuPAMAM/DNA complexes observed in the CT26 cell line appear to be encapsulated in endosome-like cytoplasmic vesicles that are not as apparent in SK-BR3 cells. Further, the concentration of these particle-containing cytoplasmic vesicles appears to increase in CT26 cells by 24 h post-transfection. For the following reasons, we postulate that these structures are in fact acidic autophagic vesicles that have accumulated in the cytoplasm of CT26 cells. First, as described previously, the confocal images taken of CT26 cells at the 1- and 4-h time points exhibit a significant preponderance of acidic cytoplasmic organelles, as made evident by the high density of yellow fluorescence in those cells (Fig. 4). This likely indicates the presence of lysosomic or acidic autophagic vesicles in the cytoplasm. Second, significant membrane ruffling is observed in the cellular TEM images taken of CT26 cells at the 4- and 24-h time-points. This ruffling, coupled with the blebbing and irregular morphologies observed in the confocal images of CT26 cells (Fig. 4), may suggest that CT26 cells are more sensitive to foreign materials than SK-BR3, and may be signs of nanomaterial induced cellular toxicity [20]. Lastly, the vesicles imaged in CT26 cells at the 24-h time point appear to contain both particle aggregates and cellular debris; this observation suggests that the cytoplasmic vesicles containing the AuPAMAM/DNA complexes may have degradative properties, or may be responsible for sequestering degraded material. Taken altogether, we believe that these vesicles are most likely autophagic in nature, though further characterizations will need to be made before the vesicles can be identified definitively.

Numerous types of nanomaterials, including PEI and PAMAM, have been previously shown to induce autophagy in various cell lines [21–24]. Though the exact role of this cellular response has not been fully characterized, many studies have postulated nanomaterial-induced autophagy to function as a means of eliminating or extruding toxic materials from the cell, and as a last-ditch mechanism for cell survival before the cell undergoes apoptosis or necrosis [25]. Typically, autophagic vesicles fuse with lysosomes to form amphisomes or autolysosomes, whereupon the contents of the fused vesicles are degraded—this specific pathway is known as autophagic flux. In autophagic dysfunction however, autophagic vesicles fail to fuse with lysosomes, resulting in an accumulation of autophagosomes and other autophagic vesicles in the cell cytoplasm. This type of accumulation is generally observed when the lysosomes have become dysfunctional, often as a result of extensive membrane permeabilization or proton pump inhibition [25]. Previous studies have demonstrated evidence of autophagosome accumulation occurring in a number of cell types, and in response to a variety of nanomaterials, including PAMAM [24, 26]. Furthermore, PAMAM and other polycationic particles have both been shown to induce lysosomal dysfunction in a number of cell types, through the “proton-sponge” mechanism described above [19]. Given our previous hypothesis regarding mucin-induced PEI and AuPAMAM aggregates causing extensive lysosomal membrane permeabilization in CT26 cells, in addition to the observation of numerous acidic, debris- and particle-containing vesicles in the cytoplasm of CT26 cells, it is possible that these same mechanisms of autophagic dysfunction are occurring in this cell type as well. These observations may explain why CT26 cells exhibit lower transfection efficiency than SK-BR3 cells, as PEI and AuPAMAM particles that have been delivered into CT26 cells become trapped in the accumulated cytoplasmic autophagic vesicles following endosomal escape, and are thus unable to traffic to the nucleus.

It is interesting to note that autophagic accumulation appears to only occur in CT26 cells, but not in SK-BR3 cells. One explanation for this observed discrepancy is that SK-BR3 cells do not undergo autophagy in response to either vector; thus, complexes that have escaped from the endosomes can freely traffic to the nucleus without autophagic interference. Lysosomal dysfunction, as previously stated, has also been demonstrated to produce increased cellular toxicity, due to the release of acidic compounds and hydrolytic enzymes from permeabilized lysosomic compartments [19]. Thus, an alternative explanation for the observed discrepancy is that the lysosomal dysfunction occurring in CT26 cells may be making these cells more vulnerable to nanomaterial-induced autophagy. Further experimentation would be required, however, to elucidate the mechanisms underlying the differential autophagic response observed in CT26 cells versus SK-BR3. In either case, given the significant membrane ruffling and blebbing observed in CT26 cells, in addition to the preponderance of autophagic vesicles visible in the CT26 cytoplasms, it is highly probable that CT26 cells are more responsive or vulnerable to foreign nanomaterials than SK-BR3 cells.

## Conclusion

Several groups have reported the lower transfectability of certain cell lines, such as CT26, without making any hypotheses as to the underlying mechanisms responsible for such phenomena [14, 15, 27]. In this work, we set out to elucidate the basis for the poor transfectability of CT26 cells compared to the easily transfected SK-BR3 cells. First, we showed that AuPAMAM- and PEI- mediated transfection is significantly more efficient in the SK-BR3 cell line than in CT26, both in terms of percent transfection and MFI (Fig. 1). Having established the large discrepancy in transfection between these two cells lines, we next quantified DNA uptake in SK-BR3 and CT26 using flow cytometry and Cy5-labeled plasmid DNA (Fig. 2). Results from these studies revealed an increased percent uptake and MFI in SK-BR3 versus CT26, and little enhancement of DNA uptake in CT26 when complexed to AuPAMAM or PEI. These results suggest that CT26 cells are more hindered in their ability to internalize DNA/vector complexes than SK-BR3 cells. Following observation of cellular uptake, we next attempted to determine the role of endosomal escape in influencing CT26 and SK-BR3 transfection efficiencies through the addition of chloroquine (Fig. 3). While treatment with chloroquine was found to improve transfection for both vectors in SK-BR3 cells, no change was observed for either vector in CT26 cells. These observations lead us to postulate that endosomal escape may be a potential area for enhancement that can be addressed to improve the transfection efficiency of PEI and other poly-cationic vectors in SK-BR3 cells; further, additional intracellular hurdles may exist beyond endosomal escape limiting transfection in CT26.

To study the intracellular trafficking pathway of complexed DNA, we established the localization of Cy5-conjugated plasmid DNA relative to organelle markers at various time points post-transfection using confocal microscopy (Figs. 4 and 5). GFP expression of the complexed DNA plasmid was higher in SK-BR3 cells than CT26 cells at all time points considered, suggesting that CT26 may be limited in its trafficking of complexes from the cytoplasm to the nucleus. Additionally, CT26 cells exhibited blebbing and irregular cell morphologies, indicating that this cell type may be more sensitive to foreign material than SK-BR3. Intracellular trafficking of AuPAMAM/DNA complexes was further explored using cellular TEM. The results of TEM imaging appeared to indicate the presence of a large number of autophagic vesicles in the cytoplasm of CT26 cells, perhaps implicating autophagosome accumulation as a potential mechanism for the reduced transfection efficiency of this cell type. The observation of autophagic vesicles may also support the notion that CT26 cells are more sensitive to exogenous materials than SK-BR3 cells. Taken together, our observations and results suggest that initial complex uptake and cytoplasmic trafficking to the nucleus are the two main variables limiting CT26 transfectability. Hence, addressing either of these variables may produce improvements in the transfectability of CT26 cells. Additional in-depth investigations of intracellular trafficking, vector/DNA release, and nuclear uptake of vectors in various cell types will further elucidate the mechanisms responsible for cell-dependent transfection efficiency. Our present work is the first step in identifying and characterizing the critical rate-limiting steps of transfection in different tissues, and will further aid in the design of more effective application-specific non-viral vectors.

## Methods

### Materials

25 kDa branched polyethylenimine (PEI), Tween 20, generation 4 polyamidoamine (PAMAM) dendrimers with ethylenediamine (EDA) core, diaminohexane (DAH) core, cystamine core or hydroxyl termini, and all other chemicals were purchased from Sigma Aldrich (St. Lewis, MO) or Fisher Scientific (Waltham, MA) unless otherwise stated. 1-Ethyl-3-[3-dimethylaminopropyl]carbodiimide hydrochloride (EDC) and N-hydroxysulfosuccinimide (Sulfo-NHS) were purchased from Thermo Scientific (Waltham, MA). Five nanometer citrate-stabilized colloidal gold nanoparticles (AuNPs) were purchased from Ted Pella (Redding, CA). Plasmid DNA with cytomegalovirus (CMV) promoter and enhanced green fluorescent protein (eGFP) as the reporter gene (pCMV-eGFP, 4.7 kb) were obtained from Clark Needham at Rice University [28]. SK-BR3 cell line, CT26 cell line, cell culture medium, and phosphate buffered saline (PBS) were purchased from ATCC (Manassas, VA). MES buffered saline and CT (PEG) 12 were purchased from Pierce (Rockford, IL). Amicon Ultra-15 10 and 50 kDa centrifuge concentrators were purchased from Millipore (Billerica, MA). Hydroxylamine and chloroquine were purchased from Alfa Aesar (Ward Hill, MA). NucBlue DAPI stain, ProLong Gold Antifade mounting medium, and Lysotracker were purchased from Life Technologies (Carlsbad, CA). Lab-Tek II 8-well chamber slides were purchased from USA Scientific (Ocala, FL). LabelIT Tracker Cy5 fluorescent probe for DNA labeling was purchased from Mirus Bio (Madison, WI).

### AuPAMAM synthesis

Polyethylene glycol (PEG) was added to 5 nm gold nanoparticles (AuNPs) (5 × 10^13^ particles/ml) to a final concentration of 83.33 μM in 12 ml. After 24 h, NaCl, sodium phosphate, and Tween 20 were added to the nanoparticle solution (to a final concentration of 0.1 M, 100 mM, and 0.1% v/v respectively) and allowed to incubate for an additional 24 h. Next, excessive PEG was removed by spinning the solution in a centrifuge filter (10,000 molecular weight cutoff) at 2500*g* for 20 min, and washing the filter three times with PBS. After the final centrifugation, the AuPEG particles were resuspended in pH 4.7 MES buffer. EDC and sulfo-NHS linkers were added to a final concentration of 0.44 and 0.59 mM, and allowed to incubate for 15 min. The AuPEG particles were then added to generation 4 diaminohexane (DAH) core PAMAM dendrimers in PBS. To estimate the amount of dendrimer needed for the conjugation, a surface packing model was used [29]. After 2 h, 2 ml of 50 mM hydroxylamine (pH 7) in PBS was added to the solution. The particles were then allowed to nutate overnight to backfill any unconjugated sulfo-NHS esters. Last, the solution was washed 3 times using a centrifuge filter (50,000 MWCO) with sterile DNase-free deionized (DI) water. The AuPAMAM was resuspended in DI water and stored at 4 °C until further use. The particles were sonicated prior to each use.

### Cell culture

SK-BR3 and CT26 cells were cultured in a humidified incubator (5% CO_2_, 37 °C). The cells were suspended in McCoy’s 5A and RPMI-1640 medium, respectively, and supplemented with 10% Fetal Bovine Serum (FBS) and 1% Penicillin–Streptomycin. Complete serum-containing media was used throughout all transfection experiments.

### AuPAMAM mediated transfection

Both AuPAMAM (9.8 nM, or 5.9 × 10^12^ NP/ml) and DNA (0.8 μg) solutions were diluted in ultra pure DI water to a final volume (of 50 μl and then combined. Water was used as the solvent in order to prevent charge screening effects prior to complex formation. The final volume of the polyplexes was 100 μl per well. The polyplex solutions were gently vortexed and left to incubate for 20 min at room temperature, before being added to cells (in a 24-well plate). The resulting complexes were added directly into wells. The next day, wells were rinsed with PBS and complete media was added; 48 h later the medium was changed. At 48 h, GFP expression of all conditions was visualized using a Zeiss Axio Observer inverted microscope. Transfection efficiency was measured using flow cytometry (BD FACSCanto II). The data presented are the mean fluorescence signals for 10,000 cells.

### Fluorescently labeling DNA

The DNA was labeled according to the published Mirus LabelIT protocol. Ultra pure DI water, 10× labeling buffer, 1 mg/ml plasmid DNA and the LabelIT tracker reagent were incubated at 37 °C for 1 h. Following a brief centrifugation step, the labeled plasmid was purified by ethanol extraction, and its concentration quantified using a NanoDrop spectrophotometer.

### Chloroquine treatment

Cells were incubated with their respective vector/DNA complex (either AuPAMAM/DNA or PEI/DNA) and 1000 μM chloroquine for 3 h. After replacement of media, the cells were left to incubate for an additional 24 h before being imaged.

### Subcellular trafficking of vectors with confocal imaging

Cells were exposed to AuPAMAM/DNA complexes for 30 min, 1, 4, 24, or 48 h in 8-well chamber slides. After incubation, wells were aspirated and cells were rinsed with PBS. Lysotracker Yellow was diluted to 50 nM and 250 μl was added to the cells for 30 min. Following Lysotracker incubation, the cells were rinsed twice with PBS and complete media was added. Next, cells were fixed with 4% paraformaldehyde for 20 min. Cells were washed twice with 500 μl PBS, then one drop of NucBlue DAPI was added and incubated for 10 min. Finally, wells were aspirated and cells were resuspended in two drops of thawed ProLong Gold Mounting Media. Chamber slides were covered using coverslips and mounting media was left to cure overnight. Edges of the coverslip were sealed with nail polish. Confocal images were taken using a Nikon Ti-E widefield, inverted fluorescent microscope equipped with a Nikon A1-Rsi confocal system.

### Cellular transmission electron microscopy

Cells were exposed to AuPAMAM/DNA complexes for 30 min, 1, 4, 24, or 48 h. Following complex exposure, cells were rinsed 3 times with PBS, fixed with 2.5% formaldehyde/2.5% glutaraldehyde in 0.1 M sodium cacodylate buffer (Electron Microscopy Sciences, Hatfield, PA, USA) at room temperature, and kept at 4 °C overnight. After fixation, the samples were washed in 0.1 M cacodylate buffer and treated with 0.1% Millipore-filtered buffered tannic acid, postfixed with 1% buffered osmium tetroxide for 30 min, and stained en bloc with 1% Millipore-filtered uranyl acetate. The samples were washed several times in water, then dehydrated in increasing concentrations of ethanol, infiltrated, and embedded in Spurr’s low-viscosity medium. The samples were polymerized in a 60 °C oven for 2 days. Ultrathin sections were cut in a Leica Ultracut microtome, stained with lead citrate in a Leica EM stainer (Leica, Buffalo Grove, IL, USA), and examined in a JEOL JEM 1010 transmission electron microscope at an accelerating voltage of 80 kV. Digital images were obtained using AMT Imaging System (Advanced Microscopy Techniques Corp, Woburn, MA, USA).

## Additional files

**Additional file 1: Figure S1.** Subcellular Trafficking of Cy5-labeled PEI/DNA Complexes in SK-BR3 Cells. (A) The intracellular trafficking of Cy5-labeled GFP reporter gene plasmid DNA (shown in red) was observed in SK-BR3 cells 1-hour, 4-hours, 24-hours, and 48-hours post-transfection via confocal microscopy. Prior to imaging, acidic organelles were stained with Lysotracker (shown in yellow) and nuclei were stained with DAPI (shown in blue). Column (A) depicts 60× magnification and Column (B) depicts Nyquist zoom of the corresponding images in column (A). Column (C) exhibits overlays of the fluorescent channels with the transmission channel for the corresponding images in column (B). Scale bar is (A) 20 μm and (B), (C) 10 μm.

**Additional file 2: Figure S2.** Subcellular Trafficking of Cy5-labeled PEI/DNA Complexes in CT26 Cells. (A) The intracellular trafficking of Cy5-labeled GFP reporter gene plasmid DNA (shown in red) was observed in CT26 cells 1-hour, 4-hours, 24-hours, and 48-hours post-transfection via confocal microscopy. Prior to imaging, acidic organelles were stained with Lysotracker (shown in yellow) and nuclei were stained with DAPI (shown in blue). Column (A) depicts 60× magnification and Column (**B**) depicts Nyquist zoom of the corresponding images in column (A). Column (**C**) exhibits overlays of all fluorescent channels with the transmission channel for the corresponding images in column (B). Scale bar is (A) 20 μm and (B), (C) 10 μm.

**Additional file 3: Figure S3.** AuPAMAM and Cy5-labeled DNA Co-localization. Complexes of AuPAMAM and Cy5-labeled GFP reporter gene plasmid DNA were observed in SK-BR3 and CT26 cells 24-hours post-transfection via fluorescence microscopy (to visualize the Cy5-labeled DNA, in red) and transmission microscopy (to visualize the AuPAMAM nanoparticles, in black). Prior to imaging, nuclei were stained with DAPI (shown in blue). The fluorescent channels were merged with the transmission channel to indicate co-localization of AuPAMAM nanoparticles with Cy5-labeled DNA at 24-hours post transfection.
