# Supplementary figures and images for: A mechanistic investigation exploring the differential transfection efficiencies between the easy-to-transfect SK-BR3 and difficult-to-transfect CT26 cell lines
Source: J Nanobiotechnology. 2017 May 2;15:36. doi: 10.1186/s12951-017-0271-8 (PMC5414217; doi:10.1186/s12951-017-0271-8)

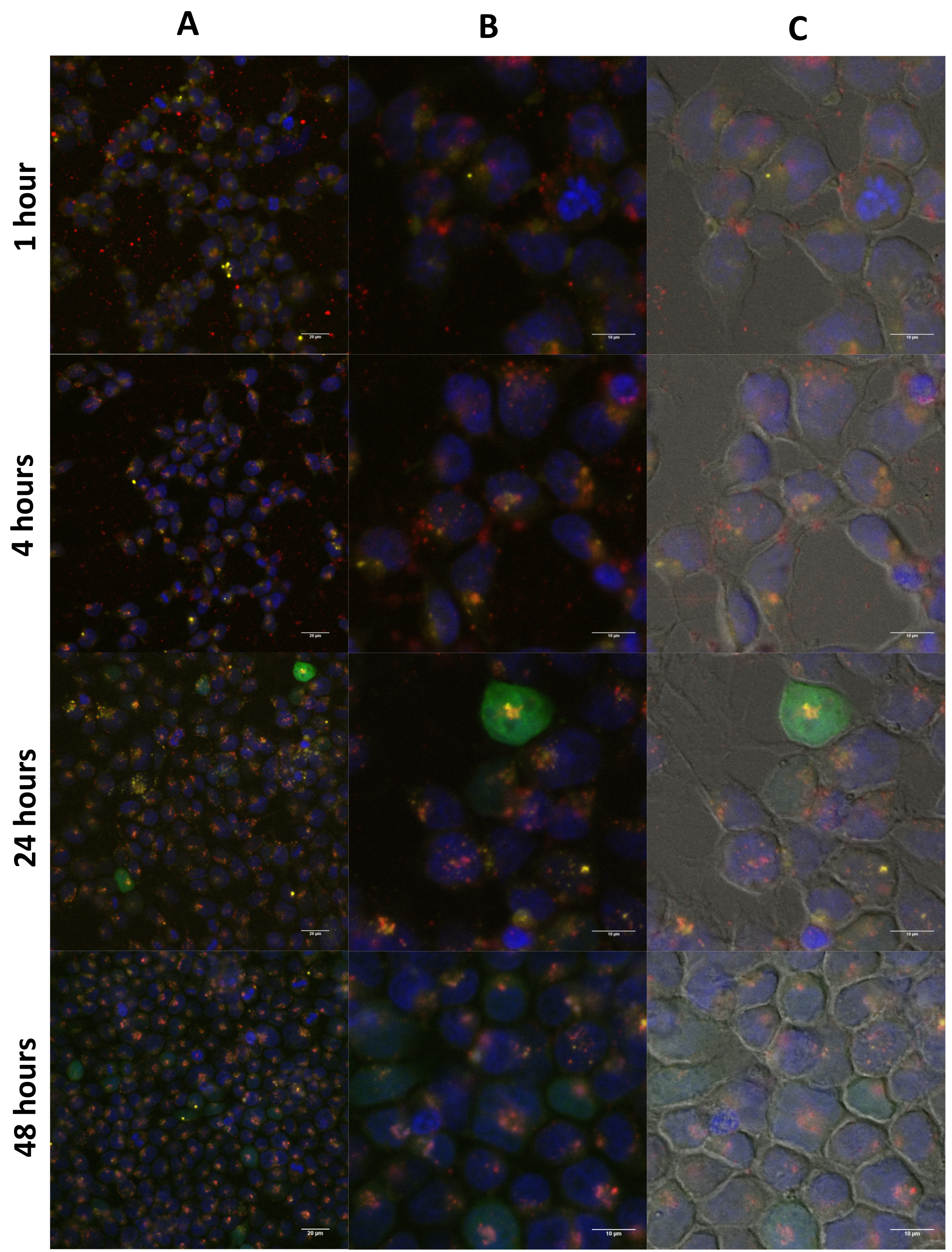

Supplement: Supplementary file 1 — Additional file 1: Figure S1. Subcellular Trafficking of Cy5-labeled PEI/DNA Complexes in SK-BR3 Cells. (A) The intracellular trafficking of Cy5-labeled GFP reporter gene plasmid DNA (shown in red) was observed in SK-BR3 cells 1-hour, 4-hours, 24-hours, and 48-hours post-transfection via confocal microscopy. Prior to imaging, acidic organelles were stained with Lysotracker (shown in yellow) and nuclei were stained with DAPI (shown in blue). Column (A) depicts 60× magnification and Column (B) depicts Nyquist zoom of the corresponding images in column (A). Column (C) exhibits overlays of the fluorescent channels with the transmission channel for the corresponding images in column (B). Scale bar is (A) 20 μm and (B), (C) 10 μm. [file 12951_2017_271_MOESM1_ESM.tif]

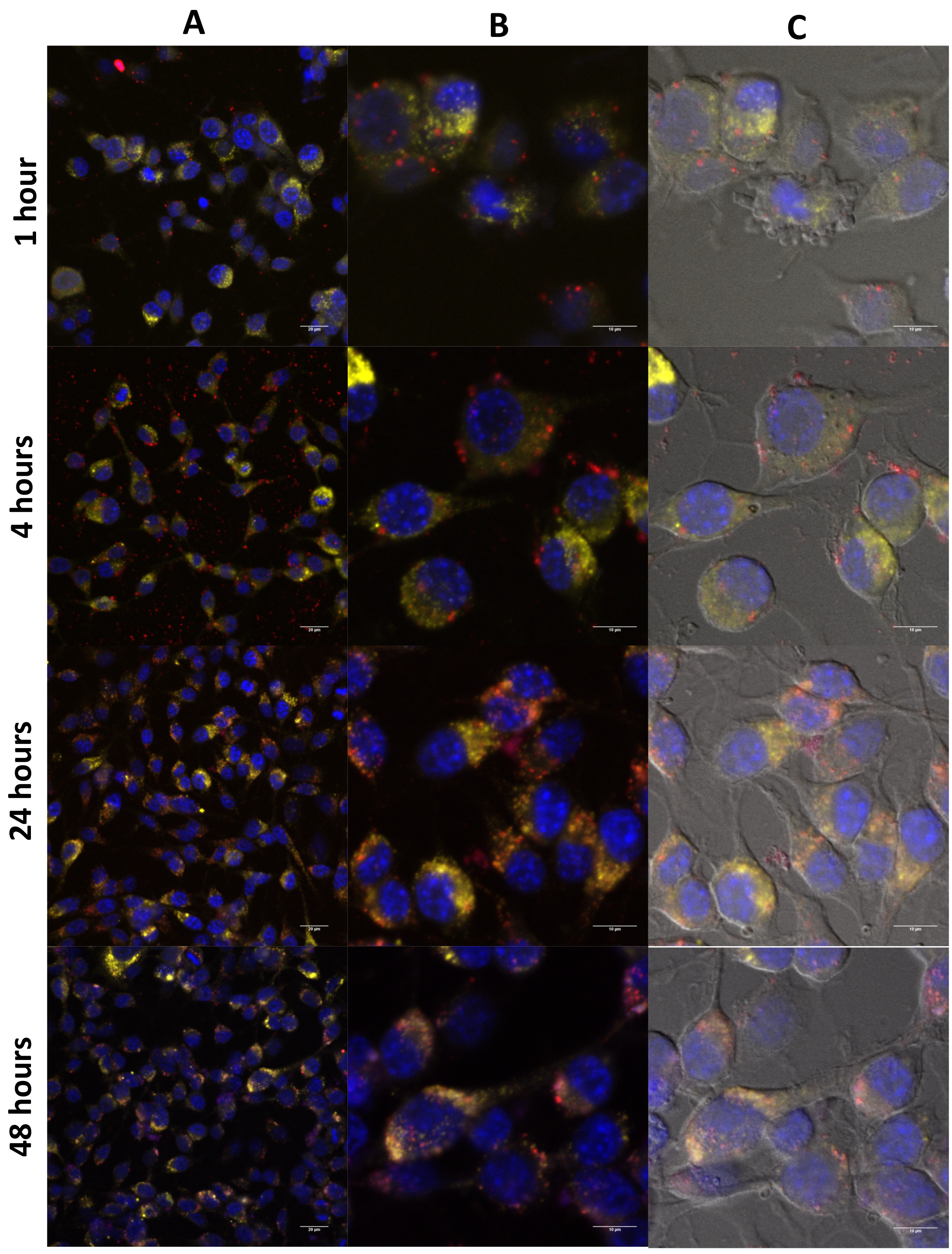

Supplement: Supplementary file 2 — Additional file 2: Figure S2. Subcellular Trafficking of Cy5-labeled PEI/DNA Complexes in CT26 Cells. (A) The intracellular trafficking of Cy5-labeled GFP reporter gene plasmid DNA (shown in red) was observed in CT26 cells 1-hour, 4-hours, 24-hours, and 48-hours post-transfection via confocal microscopy. Prior to imaging, acidic organelles were stained with Lysotracker (shown in yellow) and nuclei were stained with DAPI (shown in blue). Column (A) depicts 60× magnification and Column (B) depicts Nyquist zoom of the corresponding images in column (A). Column (C) exhibits overlays of all fluorescent channels with the transmission channel for the corresponding images in column (B). Scale bar is (A) 20 μm and (B), (C) 10 μm. [file 12951_2017_271_MOESM2_ESM.tif]

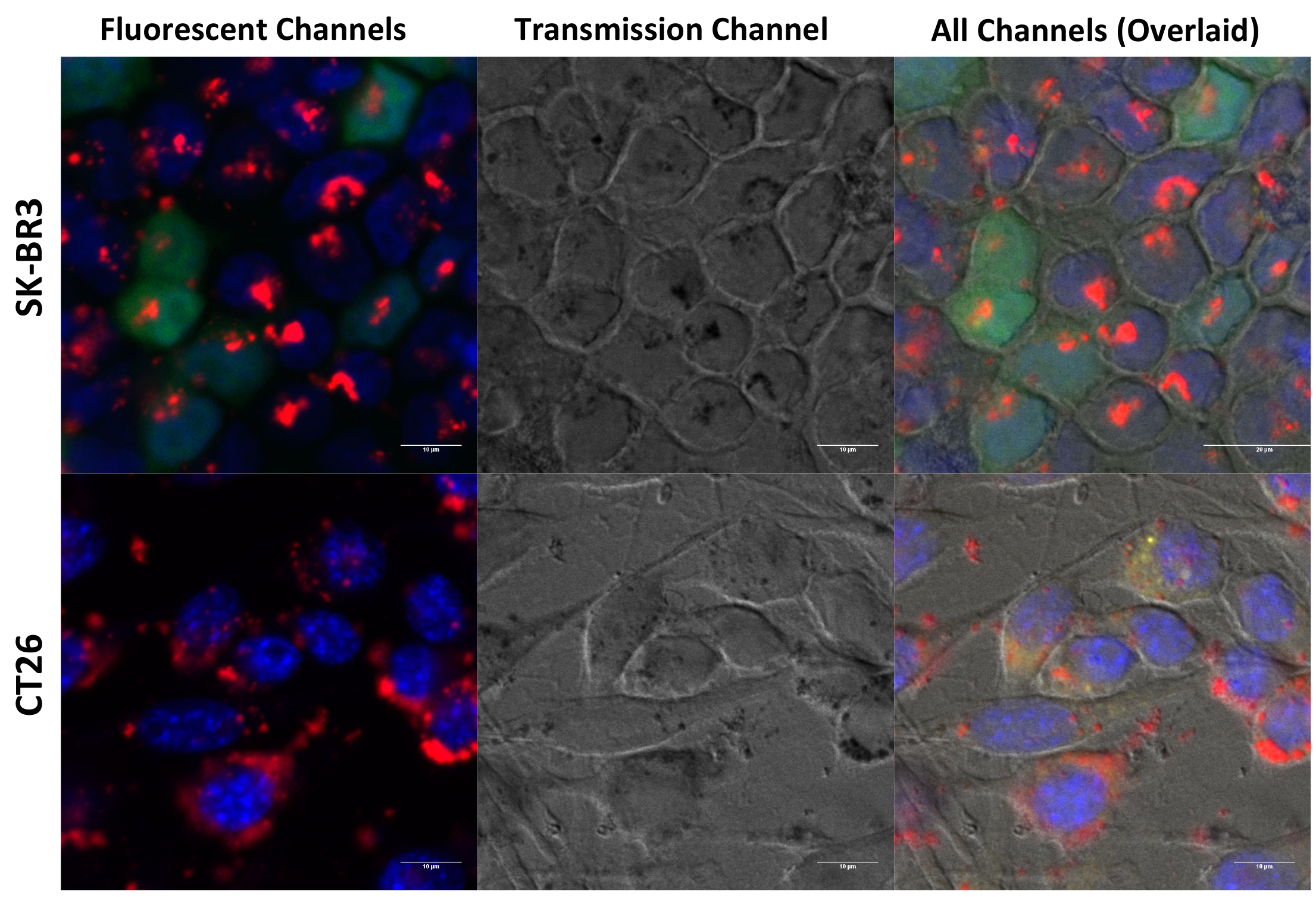

Supplement: Supplementary file 3 — Additional file 3: Figure S3. AuPAMAM and Cy5-labeled DNA Co-localization. Complexes of AuPAMAM and Cy5-labeled GFP reporter gene plasmid DNA were observed in SK-BR3 and CT26 cells 24-hours post-transfection via fluorescence microscopy (to visualize the Cy5-labeled DNA, in red) and transmission microscopy (to visualize the AuPAMAM nanoparticles, in black). Prior to imaging, nuclei were stained with DAPI (shown in blue). The fluorescent channels were merged with the transmission channel to indicate co-localization of AuPAMAM nanoparticles with Cy5-labeled DNA at 24-hours post transfection. [file 12951_2017_271_MOESM3_ESM.tif]
